# Total Bilirubin Yields Prognostic Information Following a Myocardial Infarction in the Elderly

**DOI:** 10.3390/antiox12061157

**Published:** 2023-05-26

**Authors:** Dennis Winston T. Nilsen, Peder Langeland Myhre, Svein Solheim, Sjur Hansen Tveit, Are Annesønn Kalstad, Kristian Laake, Arnljot Tveit, Ingebjørg Seljeflot

**Affiliations:** 1 Department of Cardiology, Stavanger University Hospital, 4068 Stavanger, Norway; 2 Department of Clinical Science, Faculty of Medicine, University of Bergen, 5020 Bergen, Norway; 3 Institute of Clinical Medicine, Faculty of Medicine, University of Oslo, 0315 Oslo, Norway; p.l.myhre@medisin.uio.no (P.L.M.); ssolheim@ous-hf.no (S.S.); sjur.hansen.tveit@ahus.no (S.H.T.); arekal@ous-hf.no (A.A.K.); krilaa81@gmail.com (K.L.); arnljot.tveit@vestreviken.no (A.T.); uxinlj@ous-hf.no (I.S.); 4 Department of Cardiology, Division of Medicine, Akershus University Hospital, 1474 Lørenskog, Norway; 5 Center for Clinical Heart Research, Department of Cardiology, Oslo University Hospital Ullevål, 0450 Oslo, Norway; 6 Department of Medical Research, Bærum Hospital, Vestre Viken Hospital Trust, 1346 Gjettum, Norway

**Keywords:** bilirubin, prognosis, aged, myocardial infarction, secondary prevention

## Abstract

Total bilirubin consists of an unconjugated form, solubilized by its binding to albumin, and a conjugated form representing a minor part of the circulating bilirubin. As total bilirubin in physiological concentrations is a powerful antioxidant, its concentration gradient may reflect the health status of an individual, and serve as a prognostic indicator of outcome in primary and secondary cardiovascular disease prevention. The aim of this study was to assess the association between total bilirubin and incident cardiovascular events following a myocardial infarction. Total bilirubin in serum was measured at baseline 2–8 weeks after hospitalization for an MI in 881 patients, aged 70 to 82 years, included in the OMEMI (Omega-3 Fatty acids in Elderly with Myocardial Infarction) study, where patients were followed-up for up to 2 years. The first major adverse clinical event (MACE) was the primary endpoint and consisted of nonfatal MI, unscheduled coronary revascularization, stroke, hospitalization for heart failure or all-cause death. As total bilirubin was non-normally distributed, log-transformed values and quartiles of bilirubin were analyzed using Cox regression models. The median (Q1, and Q3) baseline concentration of bilirubin was 11 (9, and 14) µmol/L, and higher log-transformed concentrations were associated with male sex, lower New York Heart Association (NYHA) class and non-smoking. MACE occurred in 177 (20.1%) patients during the follow-up. Higher concentrations of bilirubin were associated with a lower risk of MACE: HR 0.67 (95%CI 0.47–0.97) per log-unit increase, *p* = 0.032. Patients in the lowest quartile of bilirubin (<9 µmol/L) had the highest risk with HR 1.61 (95%CI 1.19–2.18), *p* = 0.002, compared to quartiles 2–4. This association remained significant even after adjusting for age, sex, body mass index (BMI), smoking status, NYHA class and treatment allocation: HR 1.52 (1.21–2.09), *p* = 0.009. Low concentrations of bilirubin (<9 µmol/L) are associated with increased nonfatal cardiovascular events or death in elderly patients with a recent myocardial infarction.

## 1. Introduction

Bilirubin is the end product of heme degradation and is essentially derived from hemoglobin, with approximately 20% being derived from other heme-containing proteins [1]. Circulating bilirubin consists of an unconjugated form bound to and solubilized by albumin, and a soluble conjugated form which represents a minor part of the total amount [1,2]. Measurement of bilirubin in its two forms is clinically useful for the differentiation of various conditions affecting its production, metabolism and excretion. Elevated bilirubin levels above the reference range will usually reflect disease or genetic disorders. However, in physiological micromolar concentrations, it possesses powerful antioxidant activity [3,4,5] and may have the potential to reduce endothelial dysfunction based on its reactive oxygen species (ROS) neutralizing antioxidant properties [2,5]. Furthermore, it promotes nitric oxide bioavailability in cardiac tissue [2,5]. Unconjugated bilirubin also possesses hormonal activity by binding to peroxisome proliferator-activated receptor alpha (PPARα), controlling target genes involved in β-oxidation [2].

Physiological levels of total bilirubin are related to future CVD events and bilirubin may serve as a prognostic marker in both primary and secondary cardiovascular disease (CVD) prevention [6,7,8,9,10,11,12]. The shape of the risk curve, whether linear, L-, U- or J-shaped, has been widely discussed [6,7,8,9,10,11,12]. Reports on the prognostic utility of bilirubin in elderly patients are scarce in primary prevention [13], and are seemingly lacking in secondary CVD prevention. In order to assess the association between circulating bilirubin and cardiovascular risk in elderly post-myocardial infarction (MI) patients, we investigated its ability to predict a major adverse clinical event (MACE) in the OMEMI (Omega-3 Fatty acids in Elderly with Myocardial Infarction) study.

## 2. Methods

The current investigation is a substudy of the OMEMI trial [14,15], which was a multicenter, placebo-controlled, double-blind clinical trial, evaluating the effect of a daily intake of 1.8 g n-3 PUFA vs. a corresponding amount of corn oil (placebo). 

Patients were enrolled at four Norwegian hospitals, including the Department of Cardiology, Oslo University Hospital Ullevål; the Department of Cardiology, Division of Medicine, Akershus University Hospital, Lørenskog; the Department of Medical Research, Vestre Viken Hospital Trust, Bærum Hospital, Gjettum; and the Department of Cardiology, Stavanger University Hospital, Stavanger, Norway. The study was organized by the Center for Clinical Heart Research at the Oslo University Hospital, Oslo, Norway, complying with the Good Clinical Practice (GCP) rules, and in accordance with the declaration of Helsinki, following the approval by the Regional Committee for Medical and Health Research Ethics. All participants provided written informed consent and the trial was registered at ClinicalTrials.gov (NCT01841944), posted 29 April 2013. Design and methods have previously been published [15]. A Case Report Form (CRF) was used for collecting information, and adverse events were assessed by a Data and Safety Monitoring Board (DSMB).

The trial primary endpoint was a composite of MI, unscheduled coronary revascularization, stroke, hospitalization for heart failure or all-cause death, and shortened MACE (Major Adverse Clinical Event). In total, 1027 participants aged 70–82 years were included 2 to 8 weeks after their index MI, whereas the current study was limited to 881 subjects with available total bilirubin measurement at baseline. 

Venous blood samples were drawn at study inclusion (baseline), i.e., 2–8 weeks after index MI, in fasting condition and before intake of morning medication (08:00–11:30 AM). Serum was prepared from whole blood that was allowed to clot and centrifuged within one hour at room temperature and 2000× *g* for 10 min. Bilirubin concentrations were analyzed using the Cobas 8000 Analyzer (Roche Diagnostics, Basel, Switzerland) in the core laboratory at each hospital. Median time between the index MI and time of blood sampling was 35 (Q1 27, Q3 45) days. 

### Statistics

Normally distributed continuous variables are presented as mean ± SD. Skewed variables are presented as median (quartile 1 [Q1], and quartile 3 [Q3]). For group comparison, ANOVA was used for normally distributed continuous variables, the Kruskal–Wallis test for non-normally distributed variables and the chi-square test for categorical variables. Clinical characteristics are presented by quartiles (Q) of total bilirubin concentrations, and compared for trend across Qs using linear and logistic regression. As total bilirubin was non-normally distributed, log-transformed values were used in regression models. 

Continuous values and quartiles of predictor variables at inclusion were subjected to Cox regression analyses for time-to-event analysis. Treatment interactions, as well as violation of the proportional hazard assumption were tested in relation to outcome. A set of potentially confounding variables was selected a priori and included in the adjusted regression models. Two models were employed in the multivariable analysis, adjusting for (1) age, sex, body mass index (BMI), smoking, NYHA class, and treatment allocation (Model 1); and (2) addition of creatinine, HbA1c and LDL cholesterol (Model 2). Hazard ratios (HR) according to quartiles are presented with 95% confidence interval (CI). The statistical analyses were performed using the Stata Software (version 16, Stata Corp., College Station, TX, USA). Tests were applied with a two-sided significance level of 5%.

## 3. Results

The median (Q1, and Q3) baseline concentration of total bilirubin was 11 [9, and 14] µmol/L; for men 11 [9, and 14] and for women 10 [9, and 12], respectively.

Demographic, clinical and biochemical features through quartiles of log-transformed total bilirubin are presented in Table 1. In multivariable models, higher concentrations of bilirubin were independently associated with male sex, lower New York Heart Association (NYHA) class and non-smoking. 

**Figure 1:**
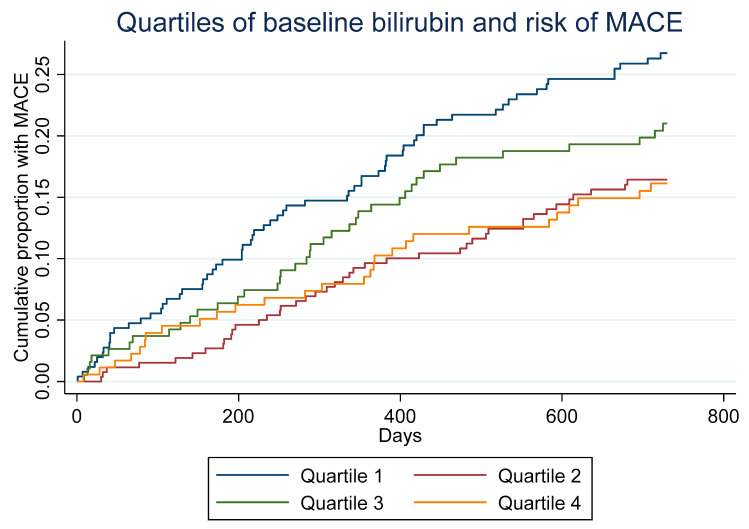
Cumulative proportion of major adverse cardiovascular events by quartiles of total bilirubin at baseline.

**Figure 2:**
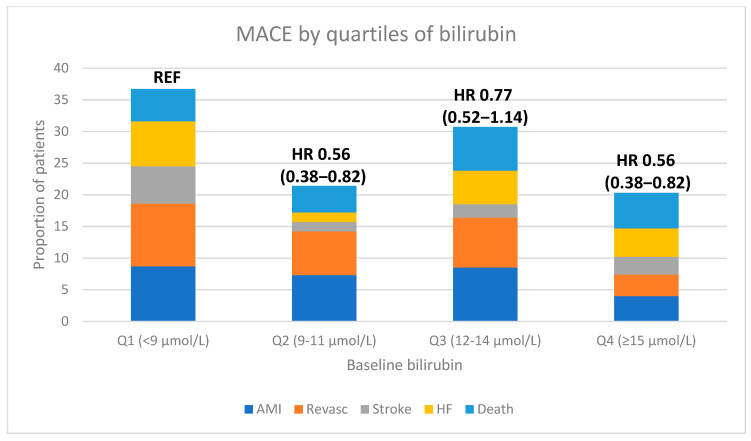
Proportion of patients with incident MACE and its components during 2 years follow-up by quartiles of baseline total bilirubin.

MACE occurred in 177 (20.1%) patients during mean ± SD 634 ± 205 days of follow-up. Higher concentrations of total bilirubin were associated with a lower risk of MACE in unadjusted analysis [HR 0.68 (95%CI 0.47–0.97) per log-unit increase, *p* = 0.032], and was close to statistical significance after adjusting for age, sex, BMI, smoking, NYHA class, and treatment allocation (Model 1) [HR 0.71 (95%CI 0.50–1.01) per log-unit increase, *p* = 0.06]. Patients in the lowest quartile of bilirubin (≤9 µmol/L) had the highest risk with HR 1.61 (95%CI 1.19–2.18), *p* = 0.002, compared to quartiles 2–4 (Figure 1). This association remained significant even after adjusting for age, sex, BMI, smoking status, NYHA class and treatment allocation (Model 1): HR 1.52 (1.21–2.09), *p* = 0.009. The individual components of the primary MACE endpoint trended in the same direction, but only reached statistical significance for coronary revascularization (Table 2; Figure 2). The association between bilirubin and MACE was consistent in women (HR 0.39 [95%CI 0.18–0.85, *p* = 0.018]) and in men (HR 0.75 [95%CI 0.50–1.12], *p* = 0.16), with a *p* = 0.13 for interaction by sex.

Risk discrimination was also performed by comparing the quartile with the highest risk (Q1) to the quartile with the lowest risk of MACE (Q4), yielding an unadjusted HR of 1.77 (95%CI 1.14–2.77), *p* = 0.011. After adjustment according to Model 1, the HR was 1.76 (95% CI 1.12–2.78), *p* = 0.014. Following additional adjustment for creatinine, HbA1c and LDL cholesterol (Model 2), the HR of total bilirubin in Q1 as compared with Q4 for the occurrence of MACE was statistically weakened [1.51 (95%CI 0.94–2.42), *p* = 0.089]. 

Total bilirubin did not correlate with creatinine (*r* = −0.02, *p* = 0.53), an established risk factor for atherosclerotic disease, and was only weakly correlated with metabolic risk factors, including HbA1c (*r* = −0.09, *p* = 0.01) and LDL cholesterol (*r* = −0.09, *p* = 0.01). Total bilirubin was found to be only weakly correlated with albumin (*r* = 0.08, *p* = 0.02), the main transporter of unconjugated bilirubin, having no bearing on causality.

## 4. Discussion

Lower levels of total serum bilirubin were associated with higher cardiovascular risk in the elderly patients with a recent MI. This association persisted even after adjusting for factors, such as age, sex, BMI, NYHA class, smoking status and treatment allocation. 

Our results suggest that healthier subjects with established CVD have higher physiological levels of total bilirubin, reflecting antioxidant properties exceeding those of individuals with lower levels of bilirubin [3,4,5].

The association between bilirubin and cardiovascular risk tended to be stronger for women than men. However, there was not a significant interaction by sex on the association between bilirubin and MACE, so we can therefore not formally claim that sex is an effect modifier on this association. A higher risk in women may be due to lower bilirubin concentrations in the female population. Women as compared to men tend to have lower values of bilirubin [16], whereas a negative association between bilirubin and body mass index (reflecting cardiovascular risk) has been reported to be stronger in males [17], contrasting with the results obtained in our study. 

Clinical studies investigating the association between total bilirubin and future CVD events are mainly related to primary prevention and show a negative relationship, usually U-shaped. This was noted by Breimer and coauthors [6] in a prospective study of 7685 middle-aged British men, and by Troughton et al. [7] who performed a case–control study of 10,593 men with an interquartile bilirubin range of 5.32–12.33 µmol/L. A similar U-shaped association with CVD events was observed in a recently performed meta-analysis including 12 prospective studies with 368,567 participants and ischemic heart disease patients (IHD) [8]. According to the authors of the meta-analysis [8], there is still no definitive consensus on the association between bilirubin concentration and CVD risk. This is exemplified by a previous meta-analysis of 11 studies reporting a negative linear relationship [9]. Similarly, Chen et al. [10] reported a negative correlation between cardiovascular death and increasing total bilirubin values in 2936 asymptomatic diabetic subjects with a mean age of 62.7 years. An L-shaped association has also been reported [11]. Whether the risk profile of total bilirubin is linear or non-linear, the fact remains that this inherent biomarker is negatively associated with CVD outcome in primary prevention studies.

Studies reporting the risk profile of total bilirubin in secondary CVD prevention are rare. We found low bilirubin levels to be a strong prognostic marker, especially when comparing the lowest (Q1) and the highest quartile (Q4) of total bilirubin. After adjusting for relevant features the association with incident MACE persisted. However, after adding known inherent risk factors of atherosclerotic disease, the association between total bilirubin and risk of MACE was attenuated, but still borderline significant (*p* = 0.089). Our results, essentially based on physiological levels of total bilirubin, are in accordance with the recently published results by Cao et al. [12] who conducted an observational study of 3809 patients with a previous MI and angiography-proven CVD and found a J-shaped risk profile of total bilirubin when including pathological levels.

Elderly patients more frequently have comorbidities than younger individuals, and may therefore have different pathophysiological mechanisms driving bilirubin levels. Moreover, reports on the association between total bilirubin and CVD events in secondary prevention in the elderly are lacking. In the OMEMI study [14,15], we have focused on 70 to 82 years old subjects with a previously diagnosed MI. These patients were randomized 1:1 to treatment with either highly purified omega-3 fatty acids or corn oil 2–8 weeks post-AMI. Although no significant treatment effects on clinical outcome was observed during the 2 years follow-up [14], we included the allocation modality in the regression models.

In a primary prevention setting, Boland and coworkers [13] studied prospectively the association between bilirubin quartile and mortality in an elderly population, enrolling 2364 residents (55% women) with a median age of 72 years recruited from a geographically defined suburban neighborhood in Southern California. The participants were followed-up for 13.7 ± 6.2 years with a cumulative mortality of 56.2%. Even though the majority of bilirubin levels were within the normal range, a significant positive association between bilirubin quartiles and mortality was observed in univariate analysis, but was no longer present when adjusted for age.

Consistent with the results obtained by Boland et al. [13], total bilirubin was not found to be associated with all-cause mortality in the OMEMI subpopulation in our study when we assessed this endpoint separately. It should be noted that the number of deaths is low. However, lower total bilirubin was associated with a higher risk of the primary endpoint MACE, which consisted of MI, unscheduled coronary revascularization, stroke, hospitalization for heart failure or all-cause death, also after adjusting for age, sex, BMI, NYHA class, smoking status and treatment modality. 

It has previously been shown that levels of bilirubin increase with age [13,18] and are determined by both erythrocyte amounts and the proportion of aged erythrocytes in aging and cardiovascular diseases [18], due to which the level of circulating bilirubin increases, despite an age-related decrease in erythrocyte counts. 

As bilirubin is a very powerful inherent antioxidant associated with the outcome in primary CVD prevention, low levels may also impact on clinical outcome in secondary CVD prevention. However, it still remains uncertain if levels of bilirubin are simply an indicator of comorbidity burden.

Although we demonstrated a negative association between total bilirubin and MACE in our study, a U-shaped or J-shaped risk profile was not obtained from our data, which may be due to a smaller sample size and fewer subjects with bilirubin levels above the normal range than that of the above-referenced studies. 

Concentration ranges of bilirubin that are labeled as “hypobilirubinemic” are inconsistent [2]. Supported by the Kaplan–Meier curves generated in our study and the selected multivariable models, we postulate that a bilirubin level below 9 µmol/L (lowest quartile) may serve as indicative of “hypobilirubinemia”. This suggested threshold would be consistent with studies showing a U-shaped or J-shaped risk distribution, respectively, and might represent a cut-off value, if future management of “hypobilirubinemia” should be introduced, although higher levels of bilirubin may also be considered. 

In a secondary prevention setting, Cao et al. [12] demonstrated increased risk of CHD events in the two lower tertiles (bilirubin levels up to 14.3 µmol/L), as compared to the upper tertile of the reference range for total bilirubin. Although a total bilirubin level above 9 µmol/L may provide prognostic information, higher levels may not be practical for clinical management.

Considering the possible atheroprotective effects of bilirubin, it is of interest that increased bilirubin within the upper physiological range is associated with favorable lipoprotein changes in patients with diabetes [19], and in subjects with Gilbert’s syndrome [20]. However, genetically elevated bilirubin levels were not associated with ischemic heart disease or MI in a report focusing on the UDP-glucuronosyltransferase gene in a meta-analysis including 14,711 cases and 60,324 controls [21]. These results suggest that increased unconjugated bilirubin is not causally associated with CVD. Thus, other factors related to increased bilirubin within its physiological range need to be taken into consideration.

Thus, CVD risk related to bilirubin may reflect other underlying mechanisms, engaging components of the heme catabolic pathway upfront of glucuronidation [22] and signaling as a ligand that directly binds to peroxisome proliferator-activated receptor alpha (PPARα) [2,23]. 

Hemeoxygenase-1 (HMOX-1) is the rate-limiting and primary enzyme involved in heme degradation which protects cells from heme-induced oxidative stress [24]. It is located ubiquitously on membranes of the smooth endoplasmic reticulum and converts pro-oxidant heme into the antioxidant biliverdin, the vasodilator carbon monoxide and iron, after which biliverdin is enzymatically reduced to bilirubin by the fast-acting biliverdin reductase [24]. There is a basal increase in oxidative status with age. Whether HMOX-1 is sufficiently upregulated to meet this challenge is still a matter of dispute [25,26]. HMOX-1 deficiency is extremely rare. However, its expression varies significantly due to HMOX-1 promotor polymorphism [27].

Hence, HMOX-1 exerts potent cytoprotective and anti-inflammatory actions which may have an impact on CVD risk [28], and may be a drug target for atherosclerotic diseases [29]. In a meta-analysis, HMOX-1 gene promotor polymorphisms were found to be associated with the susceptibility to type 2 diabetes [30]. The enzymatic activity of HMOX-1 is modulated by an endless array of physiological, pathophysiological, and nonphysiological agents and conditions [31], and several mechanisms would probably be involved in its regulation in the present setting.

Investigations based on cell cultures indicate that PPARα activators, such as fenofibrate and gemfibrozil, regulate genes controlling bilirubin synthesis in vascular cells and its metabolism in hepatic cells [32], providing beneficial antiatherosclerotic effects, essentially by the upregulation of HMOX1. Furthermore, it has been demonstrated that patients with Gilbert’s polymorphism and higher bilirubin levels are less affected by metabolic disorders, such as obesity and type 2 diabetes mellitus [2,33,34].

The structure of unconjugated bilirubin shares similarities to activators of PPARα, such as fenofibrate, and both possess cardiovascular protective properties [23,35].

Low bilirubin concentrations lead to lipid accumulation and reduced PPARα and mitochondrial function [2], originally demonstrated by Stec and coworkers [23] who noted that wild-type and PPARα KO mice on a high fat diet with fenofibrate or bilirubin for seven days both signal through PPARα dependent mechanisms. Their data demonstrate that bilirubin acts as an agonist of PPARα, and that moderate increases in bilirubin may mediate protection from adiposity.

Consequently, as unconjugated bilirubin binds directly to PPARα increasing transcriptional activity, metabolic improvement may be gained by the direct use of bilirubin nanoparticles [2,36]. Whether CHD patients will profit from such treatment will have to be answered by future research.

## 5. Limitation

In the present study, only one sample for bilirubin measurement was obtained at baseline and measurements were missing in 14%, because of which 11% of MACEs were excluded from the analysis. Mean follow-up was limited to 1.7 years. 

Patients in Q2 and Q4 had a similar risk for MACE and were both lower than that of Q3, probably mainly due to low statistical power for these comparisons, whereas the analysis of bilirubin as a continuous variable clearly underscores the reduction in risk with increasing concentrations of bilirubin. Furthermore, a convincing difference in risk was obtained when comparing the lowest to the highest quartile.

As bilirubin may reflect other derangements, we chose to limit our multivariable adjustment in Model 2 to generally accepted risk variables, including creatinine, HbA1c and LDL-cholesterol. Factors related to the generation of total bilirubin were not included in the risk assessment, as the risk related to bilirubin in itself would also reflect these variables. Furthermore, as the transport capacity far exceeds the levels within the given range of bilirubin, the amount of albumin would not affect its level.

In conclusion, publications reporting on the association between total bilirubin and future CVD events in elderly patients are lacking. Hence, the OMEMI study was tailored to investigate this population. Consistent with several reports on primary prevention, we observed a negative association between bilirubin and MACE in this secondary prevention study. Thus, low levels of total bilirubin represent a grimmer prognosis in the elderly patients included in our study.

Furthermore, we have discussed some pathophysiological aspects related to the negative metabolic effects of low bilirubin levels, whilst briefly referring to future therapeutic prospects. 

## Figures and Tables

**Table 1:** Demographic, clinical and biochemical features at baseline, presented in quartiles (Q) total bilirubin. Values are given as number (%) and mean ± SD.

Range TBil µmol/L	Q1 <9	Q2 9–11	Q3 12–14	Q4 ≥15		*p*
	*N* = 253	*N* = 262	*N* = 189	*N* = 177		
TBil, µmol/L	6.7 ± 1.3	10.0 ± 0.8	12.9 ± 0.8	20.0 ± 6.2		
Age, years	74.6 ± 3.5	74.6 ± 3.6	74.7 ± 3.7	74.9 ± 3.5		0.37
Females	93 (36.8)	79 (30.2)	40 (21.2)	39 (22.0)	<	0.001
Non-Caucasian	1 (0.4)	0 (0.0)	0 (0.0)	1 (0.6)		0.83
Smoking						0.026
Current	47 (18.6)	22 (8.4)	14 (7.4)	15 (8.5)		
Previous	118 (46.6)	126 (48.1)	91 (48.1)	92 (52.0)		
Never	88 (34.8)	114 (43.5)	84 (44.4)	70 (39.5)		
BMI, kg/m^2^	26.1 ± 4.1	26.7 ± 3.9	27.3 ± 4.4	26.6 ± 3.9		0.08
SBP, mmHg	136.1 ± 18.7	135.2 ± 21.2	137.7 ± 20.0	137.5 ± 16.9		0.28
LVEF, %	49.3 ± 9.2	50.0 ± 7.8	49.1 ± 9.4	51.0 ± 7.5		0.25
NYHA						0.008
1	143 (56.7)	171 (65.3)	128 (68.1)	121 (68.4)		
2	82 (32.5)	73 (27.9)	46 (24.5)	43 (24.3)		
3	25 (9.9)	17 (6.5)	12 (6.4)	13 (7.3)		
4	2 (0.8)	1 (0.4)	2 (1.1)	0 (0.0)		
Diabetes (any)	61 (24.1)	56 (21.4)	35 (18.5)	30 (16.9)		0.049
Hyperlipaemia	126 (49.8)	119 (45.4)	74 (39.2)	79 (44.6)		0.12
Hypertension	149 (58.9)	153 (58.4)	111 (58.7)	113 (63.8)		0.35
Uremia	22 (8.7)	4 (1.5)	6 (3.2)	6 (3.4)		0.012
COPD	29 (11.5)	20 (7.6)	9 (4.8)	10 (5.6)		0.01
HF (pre)	14 (5.5)	15 (5.7)	14 (7.4)	12 (6.8)		0.45
CVD (pre)	124 (49.0)	119 (45.4)	78 (41.3)	75 (42.4)		0.10
Hgb, g/dL	13.0 ± 1.5	13.5 ± 1.4	13.9 ± 1.4	14.2 ± 1.3	<	0.001
Leukocytes	7.3 ± 2.2	7.1 ± 2.3	7.2 ± 1.8	7.0 ± 2.0		0.24
Thrombocytes	258.8 ± 73.5	237.3 ± 67.7	233.1 ± 67.6	220.2 ± 60.4	<	0.001
Creat. µmol/L	100.1 ± 58.9	91.5 ± 24.7	93.1 ± 25.2	89.7 ± 21.6		0.009
HbA1c, %	6.2 ± 1.0	6.1 ± 0.9	6.0 ± 0.9	5.9 ± 0.8		0.021
LDLc, mmol/L	2.1 ± 0.7	2.0 ± 0.7	2.0 ± 0.7	1.8 ± 0.6		0.003
HDLc, mmol/L	1.3 ± 0.4	1.3 ± 0.4	1.3 ± 0.3	1.3 ± 0.4		0.47
Trigl., mmol/L	1.3 ± 0.7	1.2 ± 0.6	1.2 ± 0.6	1.2 ± 0.5		0.03
AST, IU/L	26.0 ± 9.6	27.7 ± 10.3	29.1 ± 14.9	33.2 ± 17.2	<	0.001
ALT, IU/L	30.0 ± 17.9	32.8 ± 18.2	34.0 ± 23.1	39.8 ± 30.2	<	0.001
GGT, IU/L	46.4 ± 55.2	42.0 ± 35.0	48.8 ± 55.9	72.8 ± 133.7	<	0.001
ALP, IU/L	83.9 ± 39.2	82.2 ± 32.9	84.6 ± 35.1	89.5 ± 68.8		0.19
Albumin, g/L	41.8 ± 4.3	41.7 ± 3.8	42.5 ± 3.7	42.5 ± 3.4		0.011

TBil = Total Bilirubin. Smoking: 1 = Current, 2 = Previous, 3 = Non-smoker. BMI = Body Mass Index. SBP = Systolic Blood Pressure. HR = Heart Rate. LVEF = Left Ventricular Ejection Fraction. NYHA = New York Heart Association classification 1–4. Diabetes (any) = both type 1 and type 2. COPD = Chronic Obstructive Pulmonary Disease. HF = Heart Failure. CVD = Cardiovascular Disease. (pre) = previous. Hgb = Hemoglobin. Leukocytes and thrombocytes; cells × 10^9^/L. Creat. = Creatinine. HbA1c = Glycosylated Hemoglobin A1c. LDLc = Low Density Lipoprotein cholesterol. HDLc = High Density Lipoprotein cholesterol. Trigl. = Triglycerides. AST = Aspartate Transaminase. ALT = Alanine Transaminase. GGT = Gamma-Glutamyl Transferase. ALP = Alkaline Phosphatase. IU = International Units.

**Table 2:** Incident cardiovascular events by quartiles of total bilirubin.

	Q-1	Q-2	Q-3	Q-4	
	*n* = 253	*n* = 262	*n* = 189	*n* = 177	*p*-Values for Trend
MACE	67 (26.5%)	42 (16.0%)	40 (21.2%)	28 (15.8%)	0.025
AMI	22 (8.7%)	19 (7.3%)	16 (8.5%)	7 (4.0%)	0.12
REVASC	25 (9.9%)	18 (6.9%)	15 (7.9%)	6 (3.4%)	0.024
STROKE	15 (5.9%)	4 (1.5%)	4 (2.1%)	5 (2.8%)	0.07
HF	18 (7.1%)	4 (1.5%)	10 (5.3%)	8 (4.5%)	0.45
DEATH	13 (5.1%)	11 (4.2%)	13 (6.9%)	10 (5.6%)	0.54

Q = quartile. MACE = Major Adverse Clinical Events. AMI = Acute Myocardial Infarction. REVASC = Revascularization. HF = Heart Failure.

## Data Availability

The data presented in this study are available in the article.

## Steering Committee

Erik Berg Schmidt, Pål Smith, Dennis WT Nilsen, Arnljot Tveit, Morten Fagerland, Svein Solheim, Ingebjørg Seljeflot, Harald Arnesen.

## Data and Safety Monitoring Board

The Committee comprised three independent academics without affiliation with the trial: Tor Ole Klemsdal, Svend Aakhus Professor and Tonje Aksnes.
